# Influences of the COVID-19 pandemic and response strategies on residents’ psychological state: The survey from Hainan Island

**DOI:** 10.1371/journal.pone.0261537

**Published:** 2022-01-20

**Authors:** Jinping Zhang, Xiangli Zhou, Bing Xue, Fang Su, Jingzhong Li, Fang Li, Tong Chu, Yeqing Cheng

**Affiliations:** 1 College of Geography and Environmental Sciences, Hainan Normal University, Haikou, Hainan, China; 2 Institute of Applied Ecology, Chinese Academy of Sciences, Shenyang, Liaoning, China; 3 School of Economics and Management, Shaanxi University of Science & Technology, Xi’an, Shanxi, China; 4 College of Urban Planning and Architecture, Xuchang University, Xuchang, Henan, China; Qazvin University of Medical Sciences, ISLAMIC REPUBLIC OF IRAN

## Abstract

Mental health is a major public health issue that affects social development. This study aims to explore the psychological state of residents of Hainan Island and its influencing factors during the COVID-19 pandemic and to provide a scientific basis for the formulation of psychological counseling measures to be used after the pandemic. We used the nonprobability snowball sampling method to conduct an online survey from February 21 to February 28, 2020, and collected a sample of 533 respondents. Using a binary logistic regression model and network analysis, the psychological state of residents and the main factors were analyzed during the concentrated outbreak of COVID-19 (from January 20 to February 16, 2020). The study found that during the pandemic, 92.7% of the respondents were in a poor state of mind, and 54.2% experienced severe psychological stress. The mental state is spatially expressed as a pattern of “high in the middle and low in the surroundings.” Second, within the four-week sample, the overall psychological tension showed an inverted U-shaped trend. Respondents feeling stressed were most common in the second week, when they accounted for 87.99% of the total. Third, individual characteristics such as gender, age, fixed expenditure, and family size significantly affect the mental state. Women, the elderly, residents with fixed expenditures, and residents with large families are at greater risk of psychological stress. Finally, external factors such as the distance between residents and the location of cases and the node degree of the pandemic transmission network have a significant impact on the psychological state. However, residents in the least developed areas of Hainan Island, which are far away from active cases and have a low node degree, are more prone to psychological pressure. The government needs to pay special attention to these groups when constructing a long-term mechanism of psychological crisis intervention and increase public health resource investment in underdeveloped areas.

## Introduction

Since December 2019, the COVID-19 pandemic has developed into an international public health emergency [1, 2], and its global outbreak has been catastrophic to the people of the world. At present, the global pandemic situation is still grim. Public health emergencies can easily affect mental health and lead to psychological problems such as anxiety, depression, and post-traumatic stress disorder [3]. Compared with physiological functions, public health emergencies have more profound and long-lasting impacts on mental health [4]. The negative emotions generated by these crises will seriously weaken social cohesion and threaten people’s health and well-being [5]. Therefore, analyzing the impact of the pandemic on the popular psychological state and carrying out targeted evacuations are of great significance for protecting people’s lives and mental health while maintaining social stability.

Over the past year, the psychological crises caused by the COVID-19 pandemic have been extreme and widespread. Research on the general population, groups suffering from underlying diseases, patients’ families, medical staff, and medical students reflects the impact of the COVID-19 pandemic on the mental states of different populations. For example, Alimoradi et al. found that pandemics can cause a higher degree of psychological stress, which in turn causes more serious sleep problems [6], and the comprehensive prevalence among men was slightly higher than that among women [7]. Research by Li et al. found that under the influence of public health emergencies, ordinary people were more psychologically stressed than front-line medical staff [8]. Studies by Cowden et al. [9] and Yuan et al. [10] found that compared with the general population, patients with chronic diseases and family members of sick children showed a more anxious mental state in pandemics and had very obvious mental health problems. Norful et al. found that due to the uncertainty caused by the pandemic, insufficient medical resources, and fear of infecting family members, medical staff generally suffered from psychological anxiety and sleep disorders [11]. Sharma et al. found that during the pandemic, nearly 80% of Indian medical students were at a high or moderate level of perceived stress, and 20% of medical students had moderate or severe anxiety [12].

The mental state may fluctuate as pandemics evolve. Scholars have analyzed the changes in the popular psychological state during different pandemic development stages. For example, Yang et al. conducted a comparative study on the changes in the common mental state before and after the outbreak and found that the outbreak of the COVID-19 pandemic had a continuous and significant negative impact [13]. Wang et al. studied emotional fluctuations through two mental health surveys in the early and peak stages of the pandemic and believed that the mental state tended to ease over time [14].

The complex changes in the psychological state under the influence of public health emergencies are the result of the combined effects of individual characteristics and external factors. Individual characteristics, such as age, gender, occupation, family size, and the nature of residence, are considered to be important factors that produce psychological pressure [15–19]. Low level of cognition [20], insufficient information on pandemic prevention, history of chronic disease, and low resilience are also reasons that cannot be ignored that increase the risk of psychological disorders [21]. From the perspective of external factors, government information releases [22], social support [23], and public opinion [24] may have a significant impact on changes in people’s psychological state. In addition, studies also found that fear of the pandemic may be related to the workplace [25], and the nature of work is also related to the psychological pressure caused by the pandemic [26].

The existing analyses of the psychological state of various groups of people during the pandemic are mostly cross-sectional studies on a single time scale or region. However, the mental state during the pandemic may have certain characteristics of temporal and spatial differentiation. Most studies did not consider regional differences in mental state, which is not conducive to formulating regionally differentiated psychological counseling policies. In addition, fluctuations in the mental state may have a certain internal connection to the number of confirmed cases, but most studies have ignored the possible impact of case numbers on people’s psychological state. Therefore, this article uses Hainan Island as a case study to analyze the temporal and spatial differentiation characteristics of the mental state and its changes during the concentrated outbreak of the COVID-19 pandemic in China and then explores the influence of the main individual characteristics and external factors, including the number of confirmed cases, on the mental state. The research results can provide a theoretical basis for formulating targeted psychological counseling programs and regionally differentiated pandemic prevention and control measures.

Hainan Island is a tropical island in China that faces mainland China located across the Qiongzhou Strait, with a land area of 35,400 km^2^. As a well-known tourist destination, Hainan Island had only 168 cases during the pandemic, but the imported cases from other parts of China were as high as 84%. Moreover, those who were infected went out frequently and engaged in a wide range of activities, with an average of more than 10 different visited venues for each person. The high frequency of travel may increase the risk of infection for ordinary people and bring greater psychological pressure to local residents. In addition, Hainan Island is isolated by the sea, is socioeconomically backward, and has an uneven distribution of high-quality medical resources. In the face of a sudden pandemic, its residents’ mental state and its influencing factors may be different from those in other parts of China. In this context, we naturally think about the following questions: (1) What is the current psychological state of the residents of Hainan Island in the face of the pandemic? (2) Does this mental state have obvious characteristics of temporal and spatial differentiation? (3) What are the main factors that affect the mental state, and are the impacts of the number of confirmed cases significant? Based on this, this article conducts an online survey of residents in various cities and counties on Hainan Island to analyze their psychological status and its temporal and spatial differentiation characteristics during the outbreak of COVID-19. Then, the main variables were screened, and the influence of individual characteristics and the external environment on the mental state was explored to answer the above questions to the degree possible. The individual characteristic variables include gender, age, education level, fixed income, fixed expenditure, employment attributes and family size. The external environmental variables include the degree of community closure, the number of confirmed cases, the distance between residents and the location of cases, the node degree of the pandemic transmission network, and the node clustering coefficient of the pandemic transmission network.

## Data and methods

### Data sources

The research team carried out a questionnaire survey on the “Impact of COVID-19 Pandemic on Residents’ Mental Status” from February 21 to February 28, 2020. The main purpose was to investigate the mental state and its changes over time, as well as possible influencing factors, during the concentrated outbreak of the pandemic in China from January 20 to February 16, 2020. The data on mental state, age, gender, education level, occupation type, family size, nature of residence, and degree of community closure in the article mainly come from this social survey. Considering that we were affected by the closures caused by the pandemic, we distributed questionnaires on the “Questionnaire Star” network platform and conducted online surveys using nonprobabilistic snowball sampling methods. Before the formal investigation, members of the research team filled out the questionnaire many times to correct obvious mistakes in a timely manner. In addition, we invited three psychology experts and three sociology experts to evaluate the questionnaire to ensure its quality and scientific accuracy. First, 30 subjects who met the requirements of the research objectives were randomly selected to complete the questionnaire, and the questionnaire was improved based on their opinions. Second, the first batch of respondents forwarded the link of the improved questionnaire to other people they knew who met the requirements. Again, the second batch of respondents forwarded to a third batch of survey respondents, and so on. After all respondents submitted the questionnaire, the “Questionnaire Star” platform automatically saved the questionnaire results.

The questionnaire survey covered all 18 cities and counties on Hainan Island. A total of 558 questionnaires were collected, of which 533 were valid, thus the effective rate of the questionnaire was 95.52%. After testing, the internal consistency reliability (Cronbach’s α = 0.931) and validity (KMO test value = 0.883, and Bartlett’s test of sphericity result is p<0.001) of the questionnaire met the statistical requirements. Furthermore, using G*Power software for post hoc analysis and setting the odds ratio OR = 1.5 [27], the significance level α = 0.05, the sample size n = 533, and the statistical power of the questionnaire is calculated as power = 0.96, thus indicating that the expected effect can be achieved by the 533 samples.

In addition to the questionnaire survey data, the number of confirmed cases and their activity trajectory data used in this article come from the information release platform of the Hainan Provincial Government. The administrative division boundaries of cities and counties on Hainan Island and other basic geographic information come from the Hainan Administration of Surveying Mapping and Geoinformation.

### Research design

#### Measure of the psychological states

According to existing research, the psychological stress caused by the COVID-19 pandemic is temporary [28]. Therefore, the temporary psychological stress of Hainan Island residents under the influence of the pandemic is investigated through four aspects in this article, including whether the respondent feels psychological stress or psychological fear, whether he or she feels psychological anxiety or psychological depression, the possibility of being infected with COVID-19, and the self-assessment of psychological stress. The questionnaire asks four questions reflecting the abovementioned psychological state to assess the psychological stress of Hainan residents under the influence of the pandemic, as shown below.

*Question 1*: *Are you nervous or afraid of the current severe pandemic situation*?
*Answer*: *A*. *Yes B*. *No*
*Question 2*: *During the pandemic*, *did you feel frustrated or upset because you were unable to work or live normally*?
*Answer*: *A*. *Yes B*. *No*
*Question 3*: *Do you think you may be infected with COVID-19*?
*Answer*: *A*. *Not at all B*. *Relatively low C*. *Difficult to judge*
*D*. *Relatively high E*. *Very high*
*Question 4*: *If the maximum psychological pressure value is 100*, *into which pressure range does your psychological pressure fall in the current environment*?
*Answer*: *A*. *0–30 B*. *31–50 C*. *51–80 D*. *81–100*

Next, we assign a quantitative value to the answer to each question. For Question 1 and Question 2, if the respondent chooses “Yes”, it is scored as 1 point; otherwise, it is scored as 0 points. For Question 3, if the respondent thinks that the possibility of COVID-19 infection is “not at all” or “relatively low”, it is scored as 0 points, “difficult to judge” is scored as 1 point, “relatively high” is scored as 2 points, and “very high” is scored as 3 points. For Question 4, if the respondent’s self-assessed psychological stress value is “0–30”, it is scored as 0 points, “31–50” is scored as 1 point, “51–80” is scored as 2 points, and “81–100” is scored as 3 points.

Then, a linear weighting method was used to comprehensively evaluate the psychological status of the respondents during the pandemic. Since it is difficult to evaluate the relative importance of the four questions, the weights are set to be equal, and the mental state value is expressed as the sum of the scores of the 4 questions. Therefore, the total score of the mental state ranges between 0 and 8. The higher the score is, the more nervous the respondent.

To further analyze the evolution of the mental state, the period of the concentrated outbreak of the pandemic is broken down into four weeks, and Question 5 is asked to understand the changes in the mental state every week.

*Question 5*: *During the period from January 20th to February 16th*, *what was the difference in your emotional state*?
*1) The first week (January 20-January 26)*
*Answer*: *A*. *Calm and not worried B*. *Slightly worried*
*C*. *Nervous and afraid D*. *Fearful E*. *Numb*
*2) The second week (January 27-February 2)*
*Answer*: *A*. *Calm and not worried B*. *Slightly worried*
*C*. *Nervous and afraid D*. *Fearful E*. *Numb*
*3) The third week (February 3-February 9)*
*Answer*: *A*. *Calm and not worried B*. *Slightly worried*
*C*. *Nervous and afraid D*. *Fearful E*. *Numb*
*4) The fourth week (February 10 to February 16)*
*Answer*: *A*. *Calm and not worried B*. *Slightly worried*
*C*. *Nervous and afraid D*. *Fearful E*. *Numb*

For each subquestion, if the respondent chooses “Calm and not worried”, it is scored as 0 points, “Slightly worried” is scored as 1 point, “Nervous and afraid” is scored as 2 points, “Fearful” is scored as 3 points, and “Numb” is scored as 4 points. Similarly, the higher the score is, the more nervous the respondent.

#### Model setting for factors of the psychological state

Individual characteristics determine the cognitive level, understanding ability and stress response to external emergencies, while the influence of the external environment will exacerbate the changes in the psychological state [29]. Therefore, it is necessary to construct a model and set relevant variables to reveal the effects of individual characteristics and the external environment on the mental state. This study believes that a mental state score between 0 and 4 indicates that although these respondents have a certain degree of psychological pressure, it may not have a significant impact on their health. However, a mental state score between 5 and 8 indicates increased psychological pressure, which may cause more serious health problems. Therefore, the mental state is further classified by using 4 as the threshold. Specifically, if the mental state score is between 0 and 4, a value of 0 is assigned, and a value of 1 is assigned if the score is between 5 and 8. Since multiple regression analysis is a common method used to explore the quantitative relationship between variables and the mental state score is a dichotomous dependent variable, this study specifically uses binary logistic regression analysis to measure the impact of various factors on the mental state. The model is expressed as follows:

$$Y_{i}=\beta_{0}+\beta_{I}X_{I}+\beta_{E}X_{E}+\varepsilon_{i}$$ (1)

where *Y*_*i*_ is the psychological state of the *i*th sample during the pandemic (*i =* 1, 2, …, 533), *β*_0_ is a constant term, *β*_*I*_ and *β*_*E*_ are regression coefficient vectors, *X*_*I*_ is the individual characteristics of residents, and *X*_*E*_ is the external factor. *ε* is the random error term.

When selecting independent variables, we first use SPSS software to diagnose collinearity for all 10 variables of individual characteristics and 5 variables of the external environment to ensure that key variables are not omitted, and eliminate 3 variables that have serious multicollinearity (VIF>10)—namely, the nature of the work unit, the degree of trust in the pandemic information, and the type of occupation. Next, the Kolmogorov-Smirnov test and the Shapiro-Wilk test and collinearity diagnosis were performed on the remaining 12 variables. The variables are not in accordance with the normal distribution (p<0.001), and there is no serious multicollinearity problem (VIF<5.5), thus they can be used as independent variables to further establish a binary logistic regression model.

Setting reasonable values for the independent variables is very important to the overall effect of the regression model. Specifically, individual characteristics include 7 variables: gender (*x*_1_), age (*x*_2_), education level (*x*_3_), having a fixed income (*x*_4_), having fixed expenditures (*x*_5_), having a permanent job (*x*_6_), and family size (*x*_7_). The external environment includes five variables: degree of closure of a community (*x*_8_), number of confirmed cases (*x*_9_), distance between the respondent and the location of cases (*x*_10_), node degree (*x*_11_), and node clustering coefficient (*x*_12_). Among them, the variable of having fixed expenditures is set to 1 if the respondent has loan, rent, insurance or entrepreneurial expenditures; otherwise, it is 0. The family size variable is set to the actual number of people living in the household during the pandemic. The variable of the number of confirmed cases refers to the cumulative number of confirmed cases in the city or county where the respondent is located. The variable of the distance between the place of residence and the location of cases refers to the shortest distance between the respondent’s residence and the actual location of confirmed cases. The node degree variable refers to the sum of the in-degree and out-degree of the node where the respondent is located in the pandemic transmission network. The node clustering coefficient refers to the clustering coefficient of the node where the respondent is located in the pandemic transmission network; the calculation method is shown in Eq (4). The assignment methods of the other variables are shown in Table 1.

**Table 1:** Assignment and descriptive statistics of independent variables.

Dimensions	Variables	Variable Assignment	Median	Standard deviation	I-Q Range		
					25	50	75
**Individual Characteristics**	Gender (*x*_1_)	1 = female, 0 = male	1.00	0.49	0.00	1.00	1.00
	Age (*x*_2_)	1 = 18–19, 2 = 20–29, 3 = 30–39, 4 = 40–49, 5 = 50–59, 6 = 60 and above	3.00	1.38	2.00	3.00	4.00
	Education level (*x*_3_)	1 = elementary school and below, 2 = junior high school, 3 = senior high school or technical secondary school, 4 = college and above	3.00	1.04	2.00	3.00	4.00
	Having a fixed income (*x*_4_)	1 = Yes, 0 = No	1.00	0.49	0.00	1.00	1.00
	Having fixed expenditures (*x*_5_)	1 = Yes, 0 = No	1.00	0.49	0.00	1.00	1.00
	Having a permanent job (*x*_6_)	1 = Yes, 0 = No	0.00	0.48	0.00	0.00	1.00
	Family size (*x*_7_)	Number of people living in a family during the outbreak	4.00	1.63	3.00	4.00	6.00
**External Factors**	Degree of closure of a community (*x*_8_)	1 = completely closed, 2 = semiclosed, 3 = not closed	2.00	0.61	2.00	2.00	3.00
	Number of confirmed cases (*x*_9_)	The cumulative number of confirmed cases	15.00	17.03	6.00	15.00	39.00
	Distance between respondent and the location of cases (*x*_10_)	The shortest distance between the respondents’ residence and the place of activity of confirmed cases	4.11	5.45	1.36	4.11	6.38
	Node degree (*x*_11_)	The sum of the in-degree and out-degree of the node	6.00	6.86	3.00	6.00	18.00
	Node clustering coefficient (*x*_12_)	The clustering coefficient of the node	0.20	0.36	0.20	0.20	0.83

From the perspective of individual characteristics, residents who are female, elderly, low-educated, have no fixed income, have fixed expenditures, and have no permanent jobs are more likely to experience psychological pressure during a pandemic. A large family size implies that the respondent has more people to support, and their psychological pressure may therefore be greater during a pandemic. From the perspective of external environmental factors, the low degree of closed management in the community, the large number of confirmed cases in cities and counties, the small distance between the residence and the location of confirmed cases, the high degree of nodes and the node clustering coefficient may cause greater psychological pressure. Therefore, we assume that variables such as gender, age, having fixed expenditures, family size, degree of closure of a community, number of confirmed cases, node degree and node clustering coefficient may have a positive impact on the mental state, while variables such as education level, having a fixed income, having a permanent job, and distance between respondent and the location of cases may have a negative impact. The descriptive statistics of the independent variables are shown in Table 1.

#### Complex network analysis

The spread of the COVID-19 pandemic presents certain complex network characteristics, and the trajectories of the confirmed cases constitute a dynamically changing and directed transmission network [30–32]. That is, the pandemic transmission network has cities and counties as nodes, case flows as connecting edges, and the number of input or output cases as weights. This study constructs a pandemic transmission network according to the spatial location changes of the cases on Hainan Island to calculate two typical characteristic parameters—namely, the node degree and node clustering coefficient—as the independent variables of the binary logistic regression model to explore the influence of the trajectory of confirmed cases on the psychological state of residents.

(1) Pandemic transmission network. We use *G* (*V*, *E*) to represent the pandemic transmission network, where *V =* {*v*_1_, *v*_2_, …, *v*_*n*_} is the collection of regions passed by the cases, and *E =* {(*v*_*i*_, *v*_*j*_) | *v*_*i*_, *v*_*j*_ *∈ V*} is the collection of trajectories (edges) of the cases between regions. The weight of network *G* is the material flows carried by edge *E*, i.e., the number of input or output cases. The adjacency matrix *A* formed by the transmission network can be expressed as follows:

$$A_{n\times n}=\left[\begin{array}{ccc} a_{11} & \cdots & a_{1n}\\ \vdots & \ddots & \vdots \\ a_{n1} & \cdots & a_{nn} \end{array}\right]$$ (2)

where *a*_*ij*_ represents the case flow from region *i* to region *j*. If there is a connection between *v*_*i*_ and *v*_*j*_—that is, if there is a connecting edge—*a*_*ij*_ = 1; otherwise, *a*_*ij*_ = 0. Since the case flow within the region is not considered, the main diagonal *a*_*ii*_ is 0.

(2) Node degree. The node degree represents the importance of nodes. The degree *d*_*i*_ of a certain node *v*_*i*_ is defined as the number of nodes connected to *v*_*i*_ or the total number of connected edges containing *v*_*i*_. In this study, *d*_*i*_ is the total number of regions that have case flows with *v*_*i*_. The node degree is divided into the in-degree *d*_*in*_ and the out-degree *d*_*out*_. When *v*_*i*_ is the input node, *d*_*i*_ is the in-degree *d*_*in*_. When *v*_*i*_ is the output node, *d*_*i*_ is the out-degree *d*_*out*_. The calculation formula is as follows:

$$d_{i}=\sum_{\dot{J}=1}^{n}a_{ij}$$ (3)

where *n* is the total number of regions with confirmed cases.

(3) Node clustering coefficient. The node clustering coefficient *C*_*i*_ represents the probability of an interconnection between the adjacent nodes of node *v*_*i*_ in the network. It is used to represent the degree of node aggregation in the pandemic transmission network while reflecting the information flow of the network following:

$$C_{i}=\frac{A_{i}}{k_{i}(k_{i}-1)/2}$$ (4)

where *A*_*i*_ is the actual number of edges of adjacent nodes and *k*_*i*_ is the number of nodes adjacent to node *v*_*i*_.

### Ethics statement

This study only investigates demographic information such as gender, age, education level, work unit, occupation type, having a fixed income, having fixed expenditures, family size, trust in pandemic information, community name, etc. It does not involve personal private information such as the respondents’ names, ID numbers, telephone numbers, and detailed home addresses, so the acquired data are not identifiable. Moreover, this is an anonymous online survey, and the respondents are unable to sign physically. We ask the respondents to complete the questionnaire only after they have read and accepted the informed consent statement in the preface of the questionnaire. Therefore, this investigation does not need to be approved by the ethics committee in China.

## Results and analysis

### Attribute characteristics of the respondents

The collected survey data was used to analyze the respondents’ main socioeconomic attributes through traditional statistical analysis. We found that most of the respondents (60.23%) were women. Their ages were mainly concentrated in the range of 20 to 49 years old (70.36%), as those 18 and 19 years old accounted for 16.14% and those over 50 accounted for 13.50% of the sample. The education level was mostly senior high school, college and above (61.16%). The respondents with fixed income and fixed expenses accounted for 58.72% and 59.47% of the total, respectively, but only 36.59% of the respondents had a fixed job. Respondents mainly belonged to medium-sized (3 to 4 family members) and large-scale (more than 4 family members) households, accounting for 43.34% and 43.71%, respectively, while small-scale households with 1 to 2 persons accounted for only 12.95% of the sample. From the perspective of community management, 74.30% of the communities were fully closed or semiclosed, and 61.54% adopted semiclosed management measures. On the whole, the sample included residents of all age groups, education levels, economic conditions, and family characteristics and conformed to and was representative of the basic conditions of Hainan Island’s socioeconomic development.

In addition, among the surveyed residents, those living in rural areas, township centers, and urban centers were quite similar, accounting for 35.27%, 21.58%, and 33.21%, respectively, while those living in the suburbs were relatively few, accounting for only 9.94%. A total of 512 (96.06%) of the respondents lived in cities and counties in which there were confirmed cases. The nearest distance between a respondent and the location confirmed cases was between 0.11 km and 32.86 km, with an average of 5.22 km and a standard deviation of 5.45.

### Spatial and temporal differentiation of psychological state

**Fig 1:**
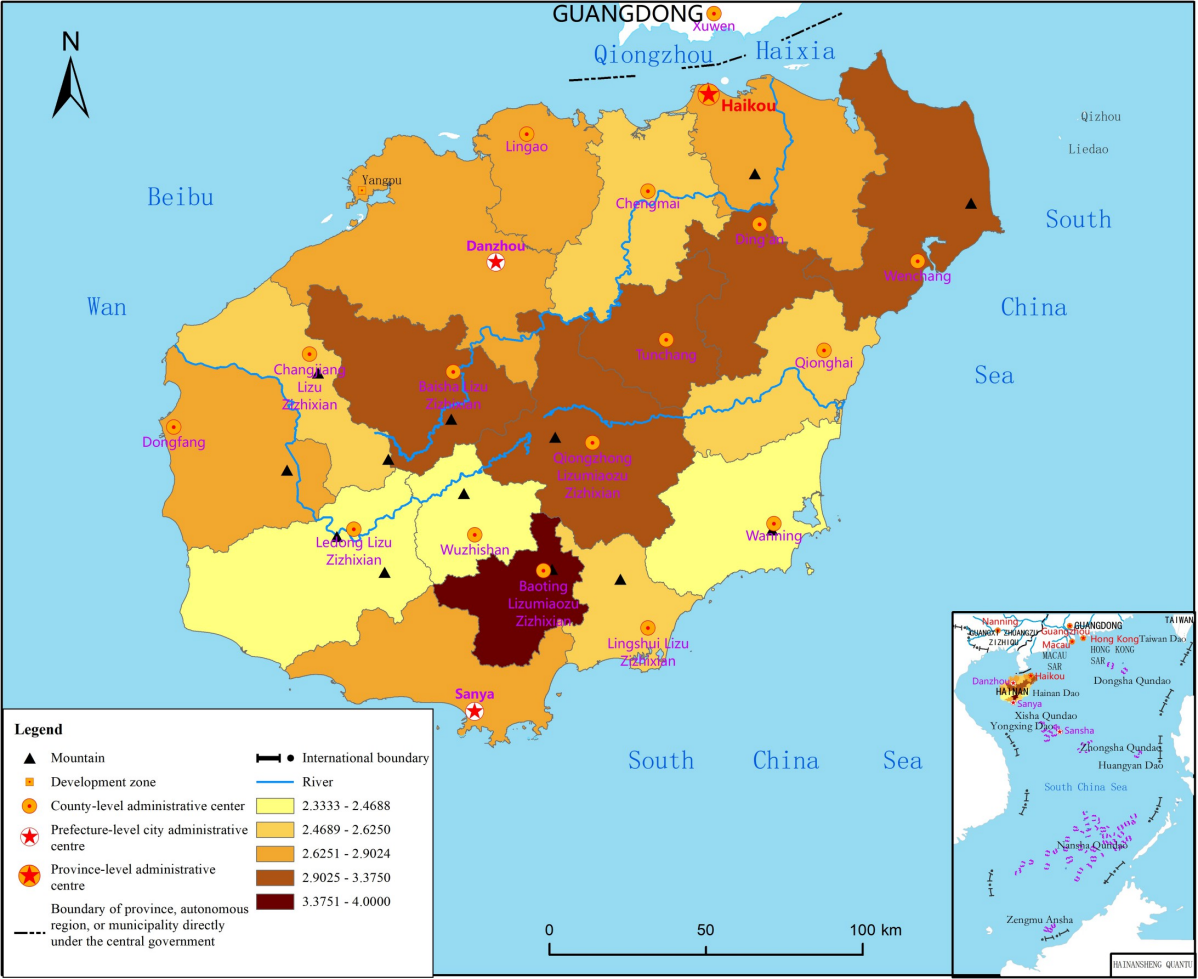
Spatial distribution of the psychological state of residents of Hainan Island. Reprinted from http://hism.mnr.gov.cn/sjkf/bzdt/201902/t20190214_3124653.html under a CC BY license, with permission from Hainan Administration of Surveying Mapping and Geoinformation, original copyright 2020.

According to the measurement rule with a total score of 8 points, the average mental state of residents on Hainan Island is 3.65. Respondents with a score between 1 and 8 account for 92.7% of the total, while those with a score greater than 4 account for 54.2%, thus indicating that the psychological state of the respondents in the face of the pandemic is generally poor. The mental state score varies greatly between regions and presents a spatial pattern of “high in the middle and low in the surroundings,” as shown in Fig 1. The central area of Hainan Island comprises minority areas with mountainous terrain and backward socioeconomic development, and the psychological state score of the surveyed residents is relatively high. Coastal cities and counties are mainly concentrated areas of the Han nationality—the main ethnic group—with plain-terraced terrain and a relatively developed economy, and the psychological state score of the surveyed residents is mostly lower than the average.

**Fig 2:**
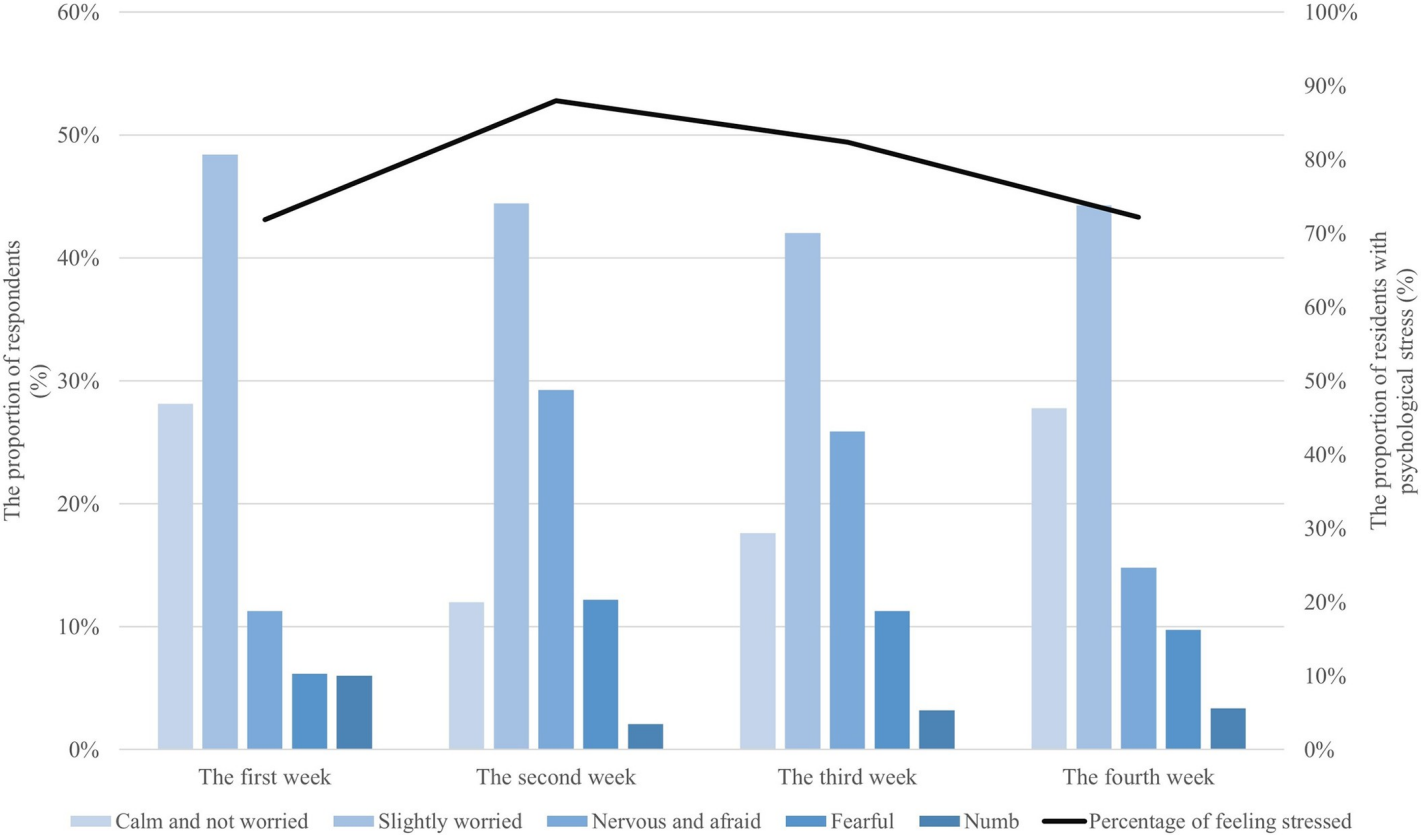
Changes in the emotional state of respondents within the four weeks.

From the perspective of changes in mental state over time, 71.86% of the respondents experienced varying degrees of psychological tension in the first week, as shown in Fig 2. In the second week, the proportion experiencing psychological stress increased to 87.99%. Beginning in the third week, the proportion of respondents who were nervous began to decline and continued to drop from 82.36% to 72.23% in the fourth week. Overall, although the emotional state of the residents was generally tense through all four weeks, the degree of emotional tension shows an inverted U-shaped characteristic for the duration of the pandemic, which fully reflects that the emotional state becomes tense in a short period of time when facing a public health emergency; as the level of awareness of the event continues to increase, the degree of tension decreases.

**Fig 3:**
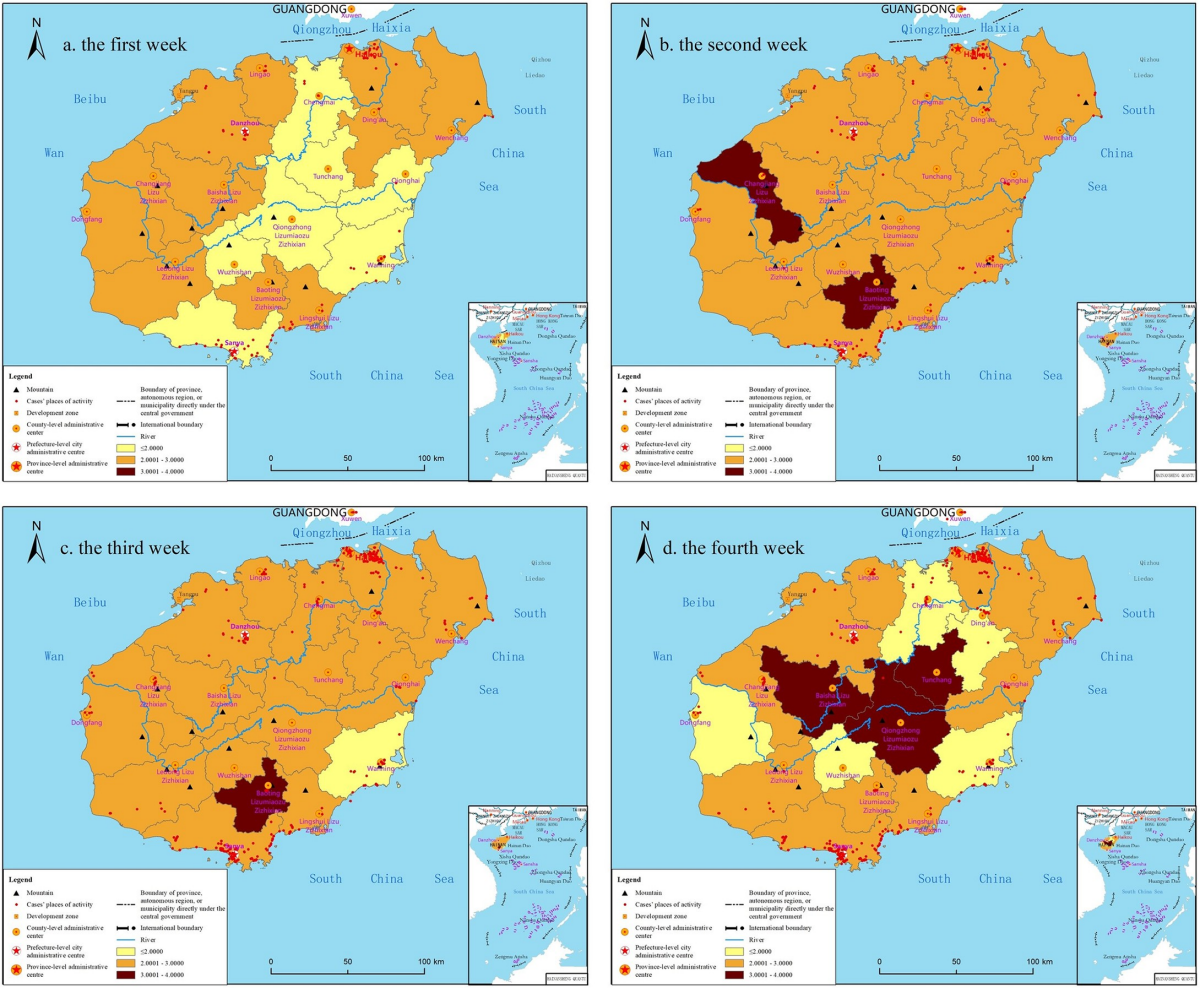
Weekly psychological state changes of residents in cities on Hainan Island. Reprinted from http://hism.mnr.gov.cn/sjkf/bzdt/201902/t20190214_3124653.html under a CC BY license, with permission from Hainan Administration of Surveying Mapping and Geoinformation, original copyright 2020.

Taking Hainan Island as a whole, the weekly mental state also shows an inverted U-shaped curve, but at the city and county scales, the weekly mental state has obvious spatial differences (Fig 3). In the first week, the mental state score in cities and counties was at medium and low levels, with an average of 2.1351. Residents with psychological stress were mainly distributed in the west and northeast regions. In the second week, the psychological state score of residents in cities and counties increased significantly, with an average of 2.4784. The psychological state score of residents in 88.9% of cities and counties was between 2.1667 and 3.0000. In the third week, the level of this score decreased slightly, with an average value of 2.4034, and that of a city in the east dropped to a low level. In the fourth week, the mental state score continued to decrease and ranged from 1.6667 to 3.1250, with an average of 2.1700, but the mental state score in the central region increased to a high level.

### Factors of the psychological state

The significance level of the binary logistic regression model is less than 0.001, thus indicating that the established model is reliable and effective. The parameter estimation results (Table 2) show that variables such as gender, age, having fixed expenditures, family size, the nearest distance between respondent and the location of cases, and the node degree have significant effects on the psychological state of residents of Hainan Island during pandemics.

**Table 2:** Parameter estimation results of the binary logistic model.

Explanatory Variables	B	Standard Error	Wald	Sig.	Exp(B) (95% CI)
**Individual Characteristics**					
** 1. Gender**	0.387*	0.211	3.348	0.067	1.472 (0.973, 2.228)
** 2. Age**	0.431***	0.099	18.878	0.000	1.539 (1.267, 1.870)
** 3. Education level**	-0.006	0.105	0.003	0.957	0.994 (0.809, 1.222)
** 4. Having a fixed income**	-0.003	0.392	0.000	0.995	0.997 (0.463, 2.149)
** 5. Having fixed expenditures**	0.532**	0.235	5.150	0.023	1.703 (1.075, 2.697)
** 6. Having a permanent job**	-0.233	0.383	0.371	0.543	0.792 (0.374, 1.678)
** 7. Household size**	0.150**	0.066	5.084	0.024	1.162 (1.020, 1.323)
**External Factors**					
** 8. Degree of closure of a community**	-0.186	0.172	1.160	0.281	0.831 (0.593, 1.164)
** 9. Cumulative number of confirmed cases**	0.014	0.010	2.268	0.132	1.015 (0.996, 1.034)
** 10. The nearest distance between respondent and the location of cases**	0.353***	0.053	45.125	0.000	1.424 (1.284, 1.579)
** 11. Node degree**	-0.044*	0.024	3.416	0.065	0.957 (0.913, 1.003)
** 12. Node clustering coefficient**	-0.471	0.419	1.264	0.261	0.624 (0.274, 1.420)
**Constant**	-2.932	0.882	11.048	0.001	0.053

Note

***, **, and * indicate statistical significance at the 1%, 5% and 10% levels, respectively.

#### The impacts of the individual characteristics

During the concentrated outbreak of the pandemic, the impact of gender on the mental state was significant at the 10% level. The estimated coefficient (0.387) indicates that female residents of Hainan Island are more prone to psychological stress, which is generally consistent with the research results of Cheng et al. [33]. The odds ratio indicates that women’s risk of psychological stress is 1.472 times that of men. Female groups have a higher degree of imagination for negative judgments in subjective reactions, which results in a more vulnerable psychological state than that in male groups under the same conditions [34]. During the pandemic, female groups who are at a disadvantage in employment will have greater psychological pressure in the face of loss of income and unemployment caused by work stoppages. In addition, females take on the important task of taking care of their families. Because the social circle is small and there are few channels through which to relieve emotions, women are prone to greater psychological stress responses under multiple pressures.

The influence of age on the psychological state of respondents is positive and significant at the 1% level. The estimated coefficient shows that for every increase in age by 1 unit, the psychological stress value of residents of Hainan Island increases by 0.431 units. The odds ratio shows that the risk of psychological stress for the elderly is 1.539 times that of the younger residents, which is basically in line with the results of Guo et al. [35]. First, most middle-aged and elderly people suffer from underlying diseases and are more likely to contract COVID-19 than young people. Therefore, they are more vulnerable and therefore prone to greater psychological pressure. Second, the cognitive ability of the elderly is low [36]. They lack accurate judgments about pandemic information and are more likely to show psychological stress reactions such as anxiety.

The variable of having fixed expenditures has a significant positive impact on the psychological state of the respondents. After the outbreak of the pandemic, Hainan Province quickly responded to the call of the Chinese government to take effective measures to control the pandemic by taking actions such as suspending work, prohibiting entering and leaving Hainan Island, cancelling public transportation between cities and counties, and restricting business in public places, but this resulted in a significant slowdown in economic development and a visible increase in the pressure on residents’ daily lives. A large number of small business owners, employees and freelancers faced a situation in which they could not make ends meet. Therefore, residents with fixed expenses such as loans, rent, insurance, and entrepreneurship costs face more severe economic pressure and are more likely to suffer from insomnia, anxiety, depression and other symptoms due to the uncertainty caused by the pandemic. The odds ratio shows that the risk of psychological stress for residents with fixed expenditures is 1.703 times that of residents without fixed expenditures.

Family size has a significant positive impact on people’s psychological state, which indicates that respondents from large-scale families are more likely to experience psychological pressure during pandemics, which is consistent with the research results of Wang et al. [14]. The odds ratio indicates that the risk of psychological stress for respondents from large-scale families is 1.162 times that of respondents from small-scale families. The questionnaire survey shows that during the pandemic period, 81.43% of Hainan Island residents exchanged information about the pandemic with their families every day. Although social topics and closed management triggered by the pandemic have strengthened family interactions, emotions have a contagious effect [37], and the spread of negative emotions within the family is more likely to increase panic. In addition, large families require additional support. Severe livelihood problems, limited living space, and seeing young children and elderly people suffering from underlying diseases will greatly increase the risk of psychological problems.

The three variables of education level, having a fixed income, and having a permanent job have a negative impact on the mental state score, but they are not significant. This shows that in the case of extremely inadequate human understanding of COVID-19 and regardless of the level of education, income or employment, people will generally experience psychological problems such as anxiety and fear due to risk factors such as pandemic confinement, economic burden, social isolation, health concerns, etc. Although the psychological pressure of respondents with low education, no fixed income, and no permanent jobs is slightly greater, there is no significant difference in the average mental state among different types of respondents.

#### The impacts of the external environment

The degree of community closure has a weak, but not significant, negative impact on the psychological state of the respondents. A high degree of closure of a community indicates a high level of local pandemic prevention and control and indicates that there are confirmed cases in the community or the community close to the place where the case is active. Residents believe that the risk of infection is high, which leads to increased psychological pressure. However, some residents believe that the fully enclosed management of the community can prevent the entrance of infected persons, which instead relieves their psychological pressure. Semienclosed and unclosed communities are located in low- and medium-risk areas with a low possibility of infection, and most residents have little psychological pressure. However, some residents believe that infected people can easily enter less enclosed communities, and psychological pressure rises instead.

**Fig 4:**
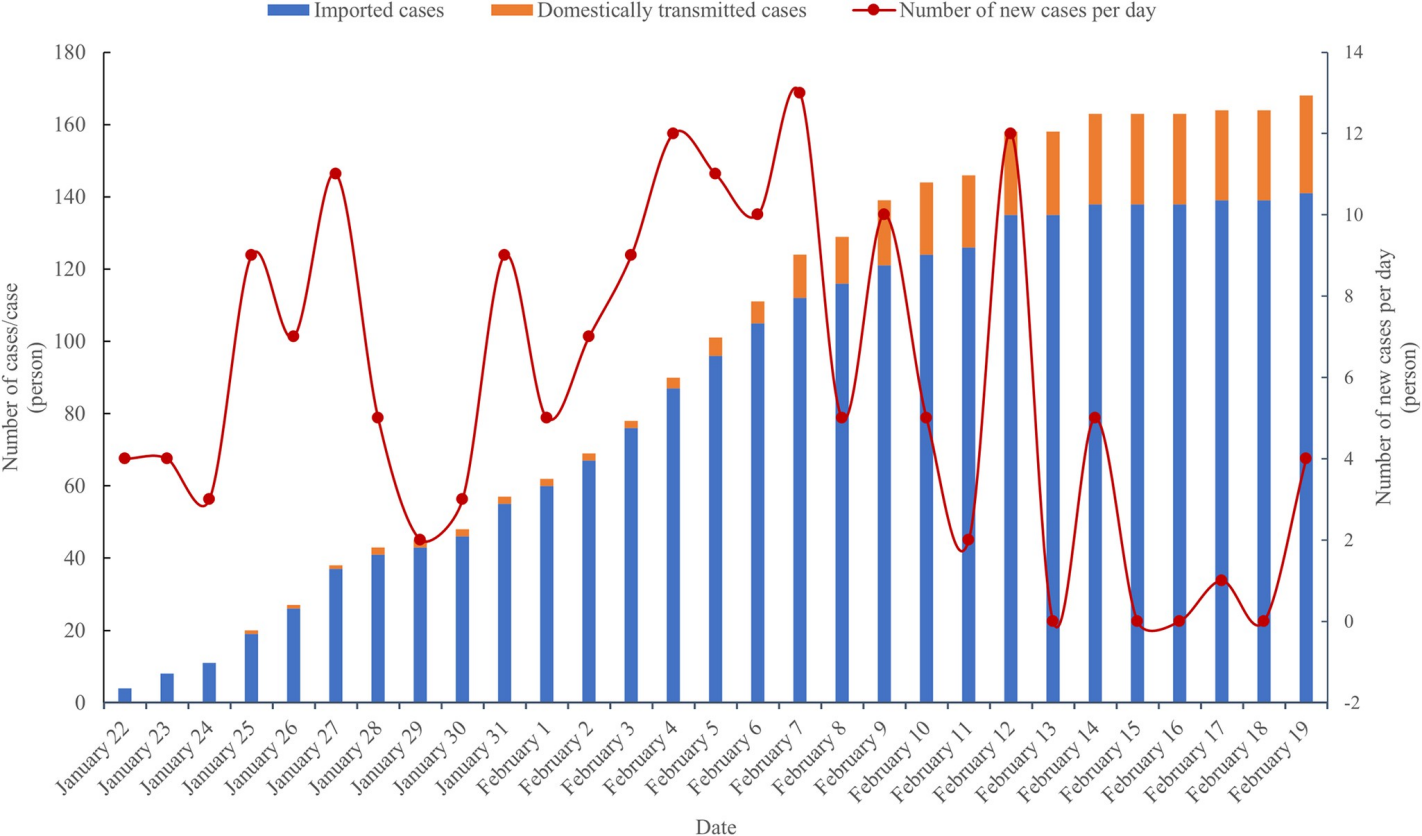
Time series changes in the number of confirmed cases on Hainan Island.

**Fig 5:**
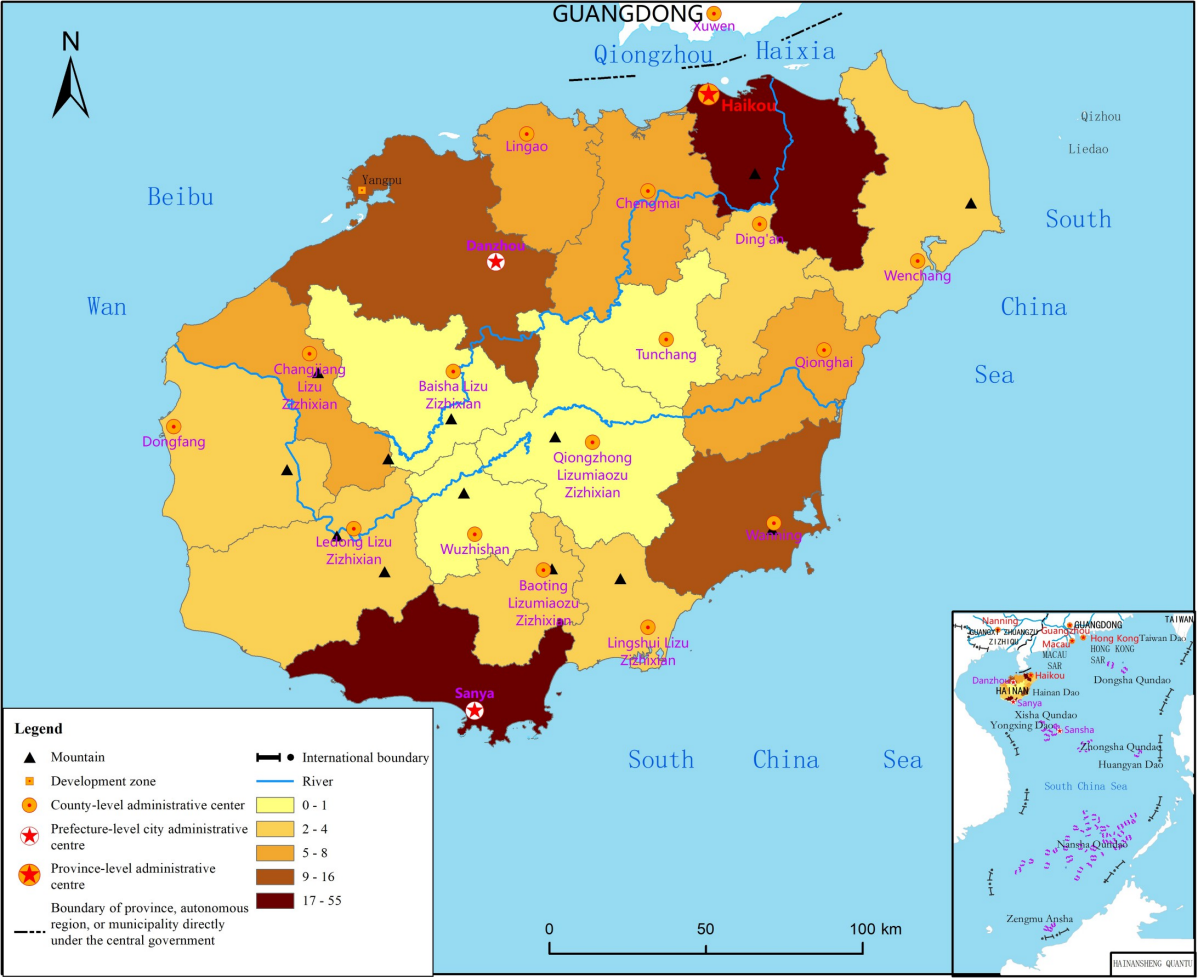
Distribution of the number of confirmed cases. Reprinted from http://hism.mnr.gov.cn/sjkf/bzdt/201902/t20190214_3124653.html under a CC BY license, with permission from Hainan Administration of Surveying Mapping and Geoinformation, original copyright 2020.

The number of confirmed cases has a weak, but insignificant, positive effect on the mental state. During the questionnaire survey, the number of new cases on Hainan Island was high in the first three weeks and dropped significantly in the fourth week (Fig 4). The cases are mainly distributed in coastal cities and counties (Fig 5). The city of Sanya at the southern end has the largest number of confirmed cases, followed by Haikou at the northern end. No confirmed cases were found in the three cities and counties on central Hainan Island. However, only two cities on Hainan Island—Haikou and Sanya—have abundant public medical and health resources with prevention and control measures already in place. Public medical resources in the central mountainous areas and other economically underdeveloped areas are in short supply, and the level of medical care is low. Given to the highly contagious nature of COVID-19 and the serious illness of the patients, the panic among the residents in the central region does not show a significant difference from that of the coastal regions on a relative basis.

The distance between residents and the location of the cases has a very significant impact on the psychological state of the residents of Hainan Island, but the relationship is not consistent with our hypothesis. The odds ratio shows that the risk of psychological stress for residents who are far away from the location of cases is 1.424 times that of residents who are near. Most of the residents of Hainan Island who are close to the location of cases are located in Sanya City and Haikou City. However, these two cities have the most developed economies, include all designated hospitals for COVID-19 treatment in Hainan Province, and have taken more effective pandemic prevention and control measures. Even if residents are close to the location of cases, their average psychological pressure is lower than that of the residents of the central region where there is no designated hospital and the medical treatment level is low.

**Fig 6:**
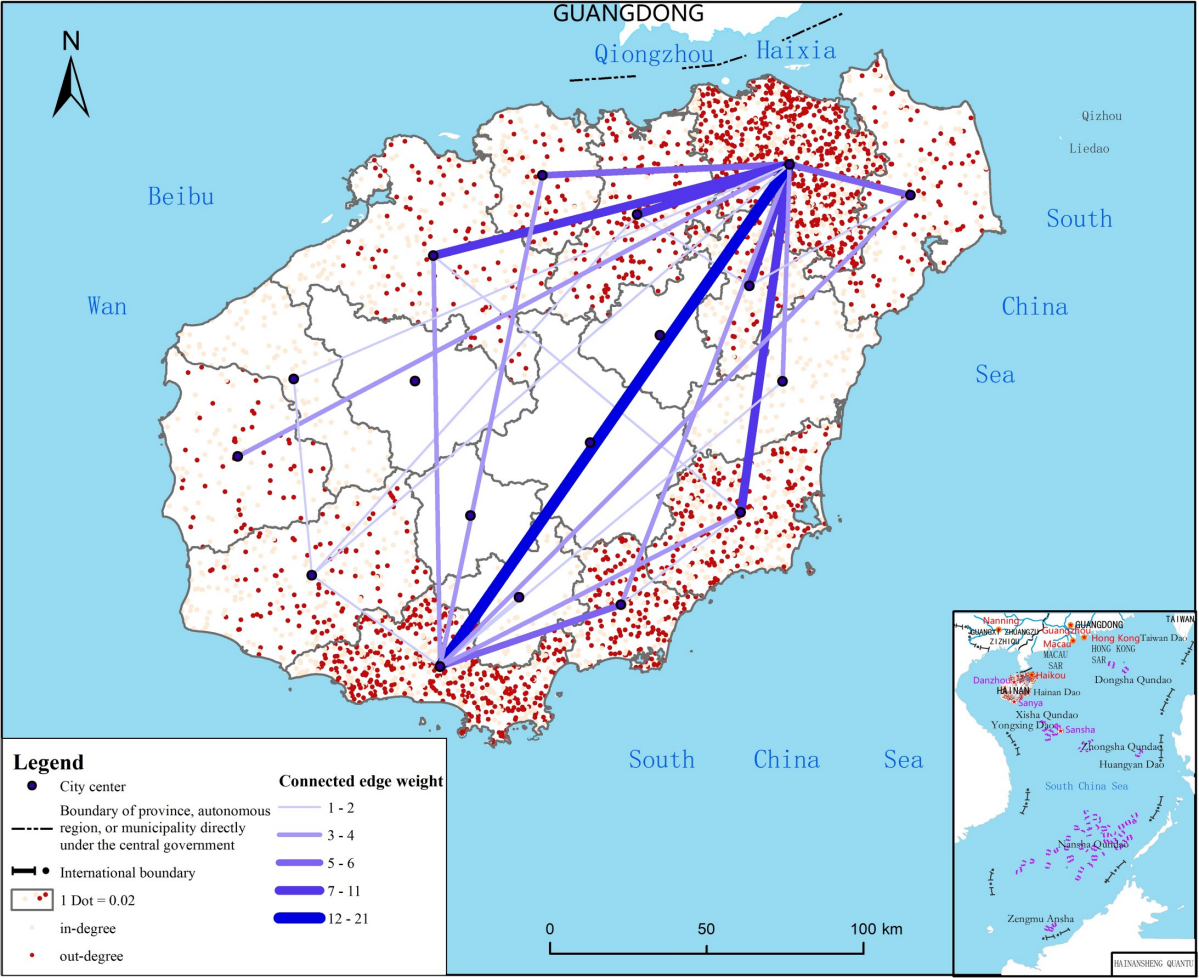
Schematic diagram of the pandemic transmission network. Reprinted from http://hism.mnr.gov.cn/sjkf/bzdt/201902/t20190214_3124653.html under a CC BY license, with permission from Hainan Administration of Surveying Mapping and Geoinformation, original copyright 2020.

The node degree of the pandemic transmission network has a significant impact on the psychological state at the 10% level, but the relationship is also not in line with our hypothesis. The odds ratio (0.957) shows that respondents with a high node degree of residence are at lower risk of psychological stress than those with a lower node degree. The input and output flows of confirmed cases on Hainan Island constitute an internal pandemic transmission network with Haikou and Sanya as the core (Fig 6). The sum of the node degree is 38, and the average score is 2.71, thus indicating that one city or county has an average case input or output interaction with three other cities or counties. However, the spatial heterogeneity of the node degree is obvious. High-value areas are mainly located in Sanya City, Haikou City and adjacent coastal cities and counties. In the two cities of Sanya and Haikou, the in-degrees are less than the out-degrees, and the sum of the weights of the outflows is much greater than that of the inflows, which means that the two cities are the sources of the cases in most other regions. In other cities and counties where there are case interactions, the in-degrees are greater than the out-degrees, and the sum of the inflow weights is low, which means that these cities and counties are mainly places where cases flow inward. Although there was no case interaction between the central region and other regions due to it having the most backward economic development and a lack of high-quality public medical services, the respondents were more worried and fearful of the pandemic than those in the coastal areas. According to the survey, nearly 70% of residents in the central region report that the main reason for their psychological tension or fear is the shortage of local medical resources. In addition, the ethnic minority areas in the central region are slightly closed, and communication with the outside world is not smooth, which intensifies the fear of the pandemic to a certain extent.

**Fig 7:**
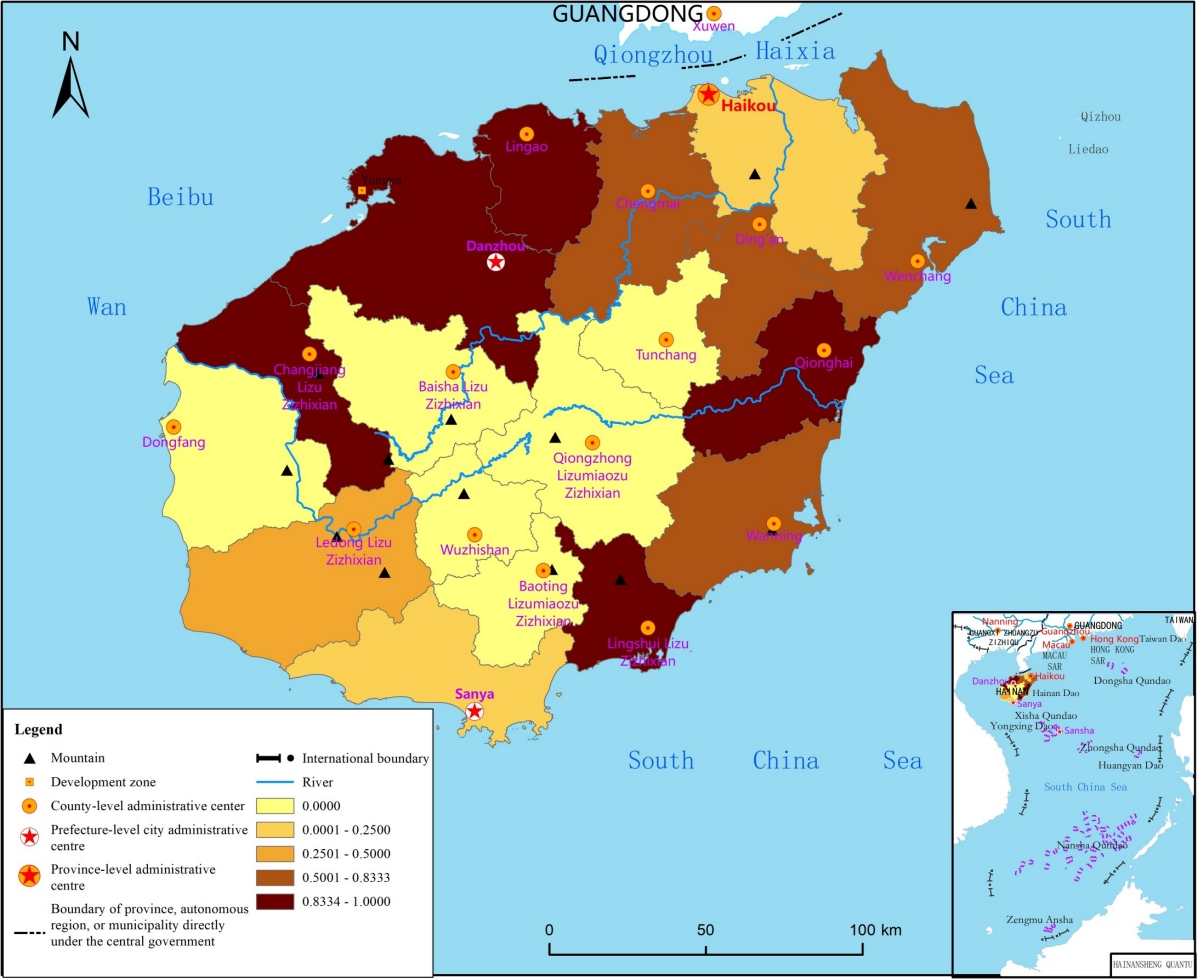
Distribution of the node clustering coefficients of the pandemic transmission network. Reprinted from http://hism.mnr.gov.cn/sjkf/bzdt/201902/t20190214_3124653.html under a CC BY license, with permission from Hainan Administration of Surveying Mapping and Geoinformation, original copyright 2020.

The node clustering coefficient has a weak, but not significant, negative effect on the psychological state of Hainan Island residents. Coastal areas with large node clustering coefficients have formed a certain spatial aggregation of information flow (Figs 6 and 7), which indicates that the cities and counties that interact with them have the risk of potential outbreaks. However, because the government has activated the highest-level emergency response for pandemic prevention and control in these areas, the risk of sustained and violent outbreaks has been effectively controlled, and the psychological state of residents has not been significantly affected.

## Discussion and conclusions

### Discussion

The family is the front line of pandemic prevention, but its importance is also the most overlooked. Under the influence of major public health emergencies, the mental status of residents of Hainan Island differed significantly between groups when divided by gender, age, fixed expenditures, family size, the distance between the resident and the location of cases, and node degree. In terms of gender, 57.3% of female respondents felt anxious or depressed, which was 7.8% higher than the percentage of males who reported similar feelings. In terms of age, 67.5% of respondents over 40 felt anxious, while only 44.2% of respondents under 30 felt anxious. In terms of fixed expenditures, 60.3% of respondents with fixed expenditures were under high psychological pressure, while only 45.4% of respondents without fixed expenditures said they felt psychologically anxious or depressed, which is a new finding. In terms of family size, 63.5% of respondents from large-scale families with more than 4 people felt anxious, in contrast to only 42.0% of respondents from small-scale households with 1 to 2 people. From the perspective of the distance between residents and the location of cases, only 37.9% of the respondents with a distance of less than 4 km indicated that they felt psychologically stressed, while 66.7% of the respondents with a distance of more than 4 km indicated that their psychological stress was high. This is also a new finding that shows the possible impact of insufficient public medical resources and low local socioeconomic levels on the psychological state of the respondents. From the perspective of the node degree of the pandemic transmission network, 58.6% of the respondents with a node degree less than or equal to 6 indicated that they had mental health problems such as anxiety and depression, while only 40.0% of the respondents with a node degree greater than 6 felt anxious or depressed, which also reflects the unique psychological state of Hainan Island residents in the face of sudden pandemics.

The community is the basic unit of social governance and the “last mile” of sanitation and pandemic prevention. In the face of major public health emergencies, the government should use the community as a unit to build a public-oriented mental health service system and a long-term psychological crisis intervention mechanism to improve its crisis management capabilities. Grassroots community governance should pay special attention to the psychological state of special groups such as women, the elderly, residents with fixed expenditures and large-scale families and carry out targeted psychological interventions and psychological consultations. Moreover, policy guarantees for these groups should be increased. For example, unemployment insurance should be applied for low-income women to enhance their ability to respond to pandemics. Other measures include: paying attention to community health services, guiding the elderly to exercise scientifically and treating underlying diseases; implementing tax cuts and fee reductions for small and micro enterprises to minimize the impact of work stoppages and reductions in consumption and services while reducing the income loss of people with fixed expenditures; and stabilizing the prices of food, basic daily necessities, and anti-pandemic supplies and providing low-income families with assistance or living allowances to ease the psychological pressure caused by family livelihood problems. Good social support can improve subjective well-being and relieve psychological pressure [38]. Therefore, a social support system for pandemics should be established, with the family as a unit, focusing on psychological counseling for residents from large-scale families, and effectively avoiding the spread of false pandemic information and panic within the family.

This study found that although the level of education has no significant effect on the mental state score, the public’s lack of basic common sense in dealing with emergencies is an important reason for its generally poor mental state. Therefore, local governments should strengthen life safety education and mental health education at the family, school, and community levels, conduct in-depth publicity and interpretation of pandemic prevention policies and measures, and generally improve the public’s psychological quality and crisis response capabilities. In addition, although there was no case activity in the underdeveloped areas of central Hainan Island and the node degree of the pandemic transmission network was small, these residents were under great psychological pressure. With the emergence of new infectious diseases, the government should support the construction of infectious disease hospitals, promote the investment of public medical resources in underdeveloped areas, and achieve equalization of public medical services to alleviate residents’ concerns about the lack of local quality medical services.

The research method has several shortcomings. First, this article measures the psychological state and its changes among residents of Hainan Island during the first concentrated outbreak of COVID-19 in China, which may not necessarily reflect the average psychological state of the Chinese public during that period. Second, the nonprobabilistic snowball sampling method is used to collect data through online questionnaire surveys, and the samples cannot represent the entire population. For example, there are too many young people, females, and highly educated residents in the sample and a small number of elderly people over 50, less educated residents, and farmers, which has a certain degree of influence on the conclusions. Finally, given that we were affected by the confinement caused by the pandemic, this article can only obtain data through online surveys. Since face-to-face interviews cannot be used to judge the accuracy of information, the data may be inaccurate.

### Conclusions

Public health emergencies can trigger a psychological stress response. After the outbreak of COVID-19, it is necessary to establish a timely and effective psychological pandemic prevention mechanism based on the characteristics of temporal and spatial changes in the mental state and their influencing factors. Based on online questionnaire surveys and confirmed case data, this article analyzes the temporal and spatial differentiation characteristics and main influencing factors of the psychological state of Hainan Island residents during the concentrated outbreak of COVID-19. The research results can provide a decision-making basis for the construction of psychological crisis prevention and intervention systems to prepare for similar emergency crisis events in the future.

According to questionnaire statistics, within one month of the COVID-19 outbreak, 92.7% of the surveyed residents experienced varying degrees of psychological stress, and 54.2% of them experienced severe psychological stress. From a spatial point of view, the mental state score presents a pattern of “high in the middle and low in the surroundings”. The psychological pressure on the residents of the underdeveloped mountainous areas in the central region is greater than that in the surrounding developed coastal areas. In terms of time, the level of psychological stress showed an inverted U-shaped characteristic within the four weeks. The number respondents who felt nervous and afraid was the highest in the second week, accounting for 87.99%, and then gradually eased, reflecting the effectiveness of pandemic prevention and control measures. In the face of sudden pandemics, the psychological state of Hainan Island residents is affected by individual characteristics and the external environment. The results of the binary logistic regression model show that women, the elderly, residents with fixed expenditures and large families are more likely to feel psychological anxiety, depression or fear, and the risk of psychological stress is 1.162 to 1.703 times that of other groups. After incorporating variables that reflect the activities of confirmed cases into external factors, it is found that two variables—the distance between resident and the cases’ place of activity and node degree—have a more significant impact on the mental state compared with the degree of community closure, number of confirmed cases, and node clustering coefficient. Residents who are far away from the location of cases and have a small node degree face greater psychological pressure, which reflects that the severely uneven allocation of medical resources and the underdeveloped level of social and economic development may have a huge impact on the psychological state of residents in the event of a major public health incident. Therefore, the government and community management departments should pay more attention to women, the elderly, families with fixed expenditures, large-scale families, and residents in underdeveloped areas when building a long-term psychological crisis intervention mechanism to minimize the psychological interference and possible psychological harm of pandemics on the public.

## Supporting information

S1 Data (XLSX)
